# CAPRIN1 Enhances Chemoresistance and Glycolysis in Laryngeal Squamous Cell Carcinoma via Regulation of ZIC5

**DOI:** 10.1155/2022/6160539

**Published:** 2022-05-05

**Authors:** Yubo Zhang, Haizhong Zhang, Jinhui Dong, Pengxin Zhao, Fang Hao, Haixia Han, Yanrui Bian

**Affiliations:** ^1^Department of Otolaryngology, The Second Hospital of Hebei Medical University, 215 Heping West Road, Shijiazhuang, 050000 Hebei, China; ^2^Department of Gland, The Second Hospital of Hebei Medical University, 215 Heping West Road, Shijiazhuang, 050000 Hebei, China

## Abstract

**Background:**

Cytoplasmic activation/proliferation-associated protein-1 (CAPRIN1) plays an important role in carcinogenesis, whereas its role in laryngeal squamous cell carcinoma remains unclear. This study was designed to investigate the roles of CAPRIN1 in glycolysis and chemoresistance and its underlying mechanisms in laryngeal squamous cell carcinoma.

**Methods:**

Cell viability was evaluated by using CCK-8 and colony formation assays. qRT-PCR, Western blotting, and immunohistochemistry were used to determine the expressions of target genes. Gene knockdown and overexpression cell lines were constructed by performing transfection of siRNAs and plasmids, respectively. Luciferase reporter assay, RNA pull-down, and RNA immunoprecipitation assays were applied to evaluate the RNA-protein interactions. The Kaplan–Meier analysis was performed to evaluate the relationship between gene expression and overall survival rate.

**Results:**

An elevation of CAPRIN1 was identified to be associated with chemoresistance and poor prognosis in patients with laryngeal cancer. The increase of CAPRIN1 promoted glycolysis and chemoresistance, whereas the knockdown of CAPRIN1 inhibited glycolysis and chemoresistance in laryngeal cancer cells. The underlying mechanistic investigation revealed that CAPRIN1 promoted glycolysis and chemoresistance of laryngeal cancer cells by the regulation of Zic Family Member 5 (ZIC5).

**Conclusion:**

CAPRIN1 promoted laryngeal squamous cell carcinoma glycolysis and chemoresistance by the regulation of ZIC5.

## 1. Introduction

Laryngeal cancer is a malignancy that occurs in the upper digestive and respiratory tract (1, 2). The majority (85-90%) of laryngeal cancer are squamous cell carcinomas (3). In 2018, Global Cancer Statistics reported that 177,422 people who are diagnosed with laryngeal cancer, and 94,771 laryngeal cancer-related death, which accounted for 1% of newly diagnosed cancer cases and related deaths, respectively (4). Many factors including inherited gene defects, the abuse of tobacco and alcohol, human papillomavirus infection, and nutritional deficiencies can cause laryngeal cancer (5). The laryngeal cancer stage is ranged from stage I to IV (6). When it is in the early stage, laryngeal cancer can be effectively treated with either surgery or radiation therapy. However, most patients are diagnosed when laryngeal cancer is already at an advanced stage, leading to treatment for patients to become difficult (6).

Unlike normal cells, laryngeal squamous cell carcinoma exhibits a high rate of glycolysis, which in return regulates the expression of oncogenes, the production of lactate, and the cancer intracellular environment (7). In addition, highly upregulated glycolysis is also associated with the activation of hypoxia-inducible factor-1 and overexpression of glycolytic-related enzymes, which promote the laryngeal squamous cell carcinoma cell growth and metastasis (8). Interestingly, previous studies revealed that targeting glycolytic pathways by inhibiting abnormal glycolysis are effective strategies for laryngeal squamous cell carcinoma therapy (9).

Therapy strategies for patients with laryngeal cancer are chemotherapy, radiotherapy, surgery, or a combination of these (10, 11). Platinum-based chemotherapy is the first-line treatment that is used for laryngeal cancer therapy (12, 13). However, platinum resistance is one of the major challenges in laryngeal cancer treatment and is observed in some patients with advanced laryngeal cancer who are treated with platinum-based drugs (14). Cisplatin therapy is widely used in patients with advanced laryngeal cancer or who received surgery at recurrence and achieved satisfactory effects in the initial stage (15, 16). Unfortunately, patients who initially respond to cisplatin therapy often developed acquired drug resistance after long-term exposure to cisplatin.

Cytoplasmic activation/proliferation-associated protein-1 (CAPRIN1) is a cytoplasmic protein, which is known to regulate cell proliferation and migration-related molecules (17, 18). A previous study showed that CAPRIN1 is associated with several types of cancers including hepatoma, osteosarcoma, gastric cancer, and breast cancer (18–21). For instance, Tan and colleagues reported that the upregulation of CAPRIN1 is correlated to poor prognosis in patients with liver cancer (20). Another study performed by Sabile and colleagues found that CAPRIN1 promotes the development of osteosarcoma by interacting with extracellular matrix protein (19). These studies supported the important roles of CAPRIN1 in the development of cancers. However, the roles of CAPRIN1 in laryngeal squamous cell carcinoma are still unknown. Therefore, in this study, we aimed to investigate the roles of CAPRIN1 in laryngeal squamous cell carcinoma and its underlying mechanisms.

## 2. Methods and Materials

### 2.1. Collection of Clinical Specimens

Patients who were enrolled in this study have read and signed the informed consent form. The clinical protocols have been reviewed and approved by the Institutional Review Board of The Second Hospital of Hebei Medical University. In this study, 100 patients with laryngeal cancer were enrolled. Laryngeal cancer and adjacent normal tissues (ANT) were collected for further use. The Kaplan–Meier analysis was performed to evaluate the relationship between CAPRIN1 expression mode and overall survival rate.

### 2.2. Immunohistochemistry

After specimens of laryngeal cancer and adjacent normal tissues were collected from patients with laryngeal cancer, paraffin-embedded specimens were sectioned and prepared with blocking buffer. Next, endogenous peroxidase was eliminated and a primary antibody against CAPRIN1 was added and incubated at 4°C overnight. Next, a secondary antirabbit IgG was added and hematoxylin counterstaining was performed at room temperature. The slides were observed and photographed under a microscope. The percentage of abundance for CAPRIN1 was analyzed by using ImageJ software.

### 2.3. Cell Lines and Construction of Cisplatin-Resistant Cell Lines

Nasopharyngeal epithelial cell line NP69 and laryngeal cancer lines including Hep-2 and TU-177 cell lines were purchased from ATCC. Hep-2 cells were cultured in Eagle's Minimum Essential Medium (EMEM) supplement with 10% fetal bovine serum (FBS, Gibco) at 37°C with 5% CO_2_. TU-177 cells were cultured in Dulbecco's Modified Eagle Medium (DMEM) supplement with 10% FBS at 37°C with 5% CO_2_.

Cisplatin-resistant Hep-2 cells (Hep-2/R) and TU-177 cells (TU-177/R) were established by the incubation of Hep-2 and TU-177 with stepwise increased concentrations of cisplatin as previously described method (22).

### 2.4. Construction of Knockdown and Overexpression Stable Cell Lines

Small interfering (si) RNA sequences against CAPRIN1 or ZIC5 were synthesized by GeneWiz (Suzhou, China). The sequences of siRNAs are shown as follows: si-CAPRIN1#1 : 5′-GCT GGA TGC CGT TTC TAA GTA-3′; si-CAPRIN1#2 : 5′-GTC CCA TCA AGT GAC TGG TAA-3′; si-ZIC5#1: 5′-GCA GTG ATC GGA AGA AAC AT-3′; si-ZIC5#2: 5′-CGG CCA CCC GCA CCC GCT CAA-3′. The cells (Hep-2, TU-177, Hep-2/R, and TU-177/R cells) were cultured in medium supplement with 10% FBS at 37°C with 5% CO_2_. When the cells reached a confluency of 60-70%, the cells were transfected with siRNAs.

To construct the overexpression stable cell lines, the cells (Hep-2 and TU-177 cells) were cultured in medium supplement with 10% FBS at 37°C with 5% CO_2_. When the cells reached a confluency of 60-70%, the cells were transfected with a plasmid containing the CDS sequence of CAPRIN1 (pSin-CAPRIN1) or ZIC5 (pSin-ZIC5) by using etrovirus. Besides, the cells that were transfected with an empty vector (pSin-vec) were used as a control. Next, the antibiotic selection was performed to establish stable overexpression cell lines. The primers to PCR the CDS sequence of CAPRIN1 and ZIC5 are as follows. CAPRIN1 forward primer: 5′- AAT TGA ATT CAT GCC CTC GGC CAC CAG C-3′, and reverse primer: 5′-AAT TGG ATC CTT AAT TCA CTT GCT GAG TGT-3′. ZIC5 forward primer: 5′-AAT TGA ATT CAT GGA GCC CCC TTT GA-3′, and reverse primer: 5′-AAT TGG ATC CCT AAT GTA TCG TCC GCA C-3′.

### 2.5. Glucose Uptake Assay

To determine glucose uptake, the 2-deoxy-D-glucose (2-DOG) uptake was determined as previously described method (8). In brief, cells were grown to about 90% confluence and then made quiescent by culturing in DMEM containing 0.4% FBS before the analysis of 2-deoxy-D-glucose (2-DOG) uptake. The uptake of 2-DOG was performed by incubating cells with glucose-free Krebs-Ringer phosphate buffer (KRP; in mM/L: 128 NaCl, 5.2 KCl, 1.3 CaCl2, 2.6 MgSO4, and 10 Na2HPO4) supplemented with 1% bovine serum albumin (BSA), and 10 mM HEPES, along with 2-[^3^H] DOG (1 *μ*Ci/well) and unlabeled 2-DOG for 5 min. Next, the reaction was ended by adding cold KRP containing 200 *μ*M phloretin for10 min. The radioactivity remaining in the cells was determined by a liquid scintillation counter. Cells were then lysed in buffer (10 mM Tris-HCl, pH 7.0, 150 mM NaCl, 1% Triton X-100, and 0.1% sodium dodecyl sulfate [SDS]) and protein concentrations were determined by BCA Protein Assay (Pierce).

### 2.6. Lactate Secretion Assay

After the cells were transfected, the medium was collected. The concentrations of lactate were determined by using a commercial lactate assay reagent, according to the document of the manufacturer (Abcam).

### 2.7. Determination of Cell Viability

In this study, cell viability was determined by using CCK-8 and colony formation assay, respectively. MTT assay was performed as previously described. After the cells were treated with the indicated concentrations of cisplatin, MTT reagent (10 *μ*L) was added to each well and incubated for 4 hours. Detergent Reagent was then added to dissolve the purple precipitate and incubated in dark at room temperature for another 2 hours. The plate was read at a wavelength of 570 nm. For cell colony formation assay, after the cells were treated with cisplatin for the indicated time, the cells were stained with 0.5% crystal violet solution, and the colonies were counted using a stereomicroscope.

### 2.8. Luciferase Activity Assay

To investigate the intermolecular interactions, the luciferase-activity assay was applied. The Hep-2 cells were transfected with a constructed plasmid (ZIC5 3′UTR or psi-check2). After the cells were transfected with pSin-CAPRIN1 or pSin-vec, luciferase activity was determined. In addition, the TU-177 cells were transfected with a constructed plasmid (ZIC5 3′UTR or psi-check2). After the cells were transfected with si-CAPRIN1#1, si-CAPRIN1#2, or si-NC, luciferase activity was determined.

### 2.9. RNA Pull-Down and RNA Immunoprecipitation Assays

An in vitro transcription was performed to obtain the ZIC5 mRNA. Next, the ZIC5 mRNA was labeled with biotin. Proteins were lysed from the cells and then incubated with biotinylated-ZIC5 in the presence of streptavidin Dynabeads. After the proteins were resolved, a Western blotting assay was performed to determine the expressions of CARPIN1 and *β*-actin.

RNA immunoprecipitation (IP) assay was performed according to a previously reported method (23). In brief, Sepharose beads were incubated with antibodies against CAPRIN1 or IgG. Next, the cell lysate was incubated with Sepharose beads. After that, the RNA was eluted from the beads and reverse transcription was performed. Finally, the qRT-PCR was conducted to analyze the mRNA expression levels of ZIC5.

### 2.10. qRT-PCR

The primers were synthesized by GeneWiz (Suzhou, China) and the sequences are shown as follows: CAPRIN1 sense: 5′-TCT CGG GGT GAT CGA CAA GAA-3′, and antisense: 5′-CCC TTT GTT CAT TCG TTC CTG G-3′. ZIC5 sense: 5′-GTC TAT GGG CCT GAT TGT GTA GT-3′, and antisense: 5′- GCC AAA TCC GCT AAT CTC AGC-3′. *β*-Actin sense: 5′-AGC GAG CAT CCC CCA AAG TT -3′, and antisense: 5′- GGG CAC GAA GGC TCA TCA TT-3′. Total RNA was isolated from tissues or cells by using TRIzol™ Reagent according to the manufacturers' document. Isopropyl alcohol was used to precipitate the RNA. After the supernatant was completely removed, RNA washing buffer was then added and vortexed. RNase-free water was added to elute RNA. The concentration and quality of RNA samples were determined by using the Nanodrop instrument. Reverse transcription was performed to synthesize the cDNA library prior to the advanced master mix which was applied for the quantitative analysis. A melt curve was applied to analyze the accuracy of the PCR reaction. 2-^△△^Ct values were calculated to evaluate the expression levels of each target gene. The relative expression levels of CAPRIN1 and ZIC5 were normalized to the *β*-actin mRNA expression levels.

### 2.11. Western Blotting Assay

The protein extraction and qualification were performed according to the previous reports (24, 25). The treated cells were scratched by using a radioimmunoprecipitation assay buffer containing protease inhibitor on ice. Next, the removal of the insoluble components was performed by using centrifugation. A bicinchoninic acid protein assay kit was applied to qualify protein concentration. An equal amount of protein sample (10 *μ*g) was loaded on the precast SDS-PAGE gel followed by transferring to 0.22 *μ*m polyvinylidene fluoride membrane. Membrane blocking was applied by incubating the membrane with 5% bovine serum albumin at room temperature for 2 hours. The primary antibodies against CAPRIN1 (1 : 1000), ZIC5 (1 : 1000), and *β*-actin (1 : 2000) were added to incubate with the membrane at 4°C for overnight. Next, the membrane was then incubated with horseradish peroxidase conjugated-secondary antibodies at room temperature for another 2 hours. The Biorad Gel Imaging System was applied and the expressions of target proteins were analyzed by comparing with the internal control (*β*-actin).

### 2.12. Statistical Analysis

Data analysis was performed by using Graphpad Prism8 software (San Diego, CA, United States). All data were presented as the means ± SD. Student's two-sided *t*-test or one-way ANOVA was performed and a *P* value less than 0.05 was considered a statistical difference.

## 3. Results

### 3.1. An Elevation of CAPRIN1 Was Identified to Be Associated with Chemoresistance and Poor Prognosis in Laryngeal Cancer

**Figure 1:**
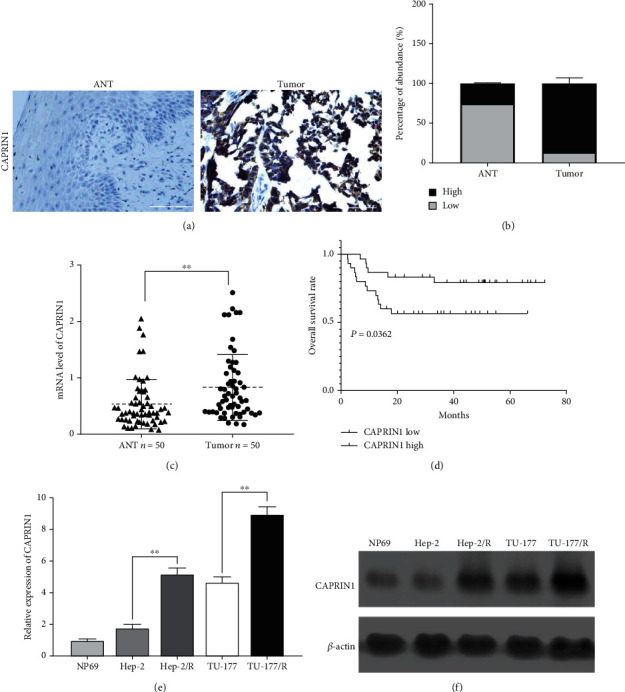
An elevation of CAPRIN1 was identified to be associated with chemoresistance and poor prognosis in laryngeal cancer. (a and b) Immunohistochemistry was used to determine the protein expressions of CAPRIN1 in laryngeal cancer tissues and adjacent normal tissues (ANT). Representative photographs were provided. Scale bars, 100 *μ*m. (c) Besides, qRT-PCR was used to determine the mRNA expression levels of CAPRIN1 in laryngeal cancer tissues and ANT. (d) The Kaplan–Meier analysis was performed to compare the overall survival rate between the CAPRIN1 high group and CAPRIN1 low group from patients with laryngeal cancer (*n* = 50). (e and f) qRT-PCR and Western blotting were applied to determine the mRNA and protein expression levels of CAPRIN1 in laryngeal cancer cell lines including Hep-2 and TU-177 and cisplatin-resistant cells including Hep-2/R and TU-177/R as well as nasopharyngeal epithelial cells (NP69). ^∗^*P* < 0.05; ^∗∗^*P* < 0.01.

We investigated the expressions of CAPRIN1 in laryngeal cancer and ANT tissues and found that the expressions of CAPRIN1 in laryngeal cancer tissue were significantly higher than those in the ANT tissue (Figure 1(a)). Supportively, the percentage of the abundance of CAPRIN1 in laryngeal cancer tissue was also higher than that in the ANT tissue (Figure 1(b)). In addition, we observed a significant elevation of the mRNA expression levels of CAPRIN1 in laryngeal cancer tissue (Figure 1(c)). In addition to investigating the expressions of CAPRIN1, we also evaluated the relationship between CAPRIN1 expression and overall survival rate in patients with laryngeal cancer. We found that patients with CAPRIN1 low expression mode had a lower overall survival rate as compared to the patients with CAPRIN1 high expression mode (Figure 1(d)).

Next, we investigated the relationship between CAPRIN1 and cisplatin resistance. Our results showed that the relative mRNA expression levels of CAPRIN1 were significantly increased in the cisplatin-resistant cell lines (Hep-2/R and TU-177/R) (Figure 1(e)). Consistently, the protein expressions of CAPRIN1 in cisplatin-resistant cell lines were higher than those in laryngeal cancer cell lines (Figure 1(f)).

### 3.2. CAPRIN1 Promoted Glycolysis and Chemoresistance in Laryngeal Cancer Cells

**Figure 2:**
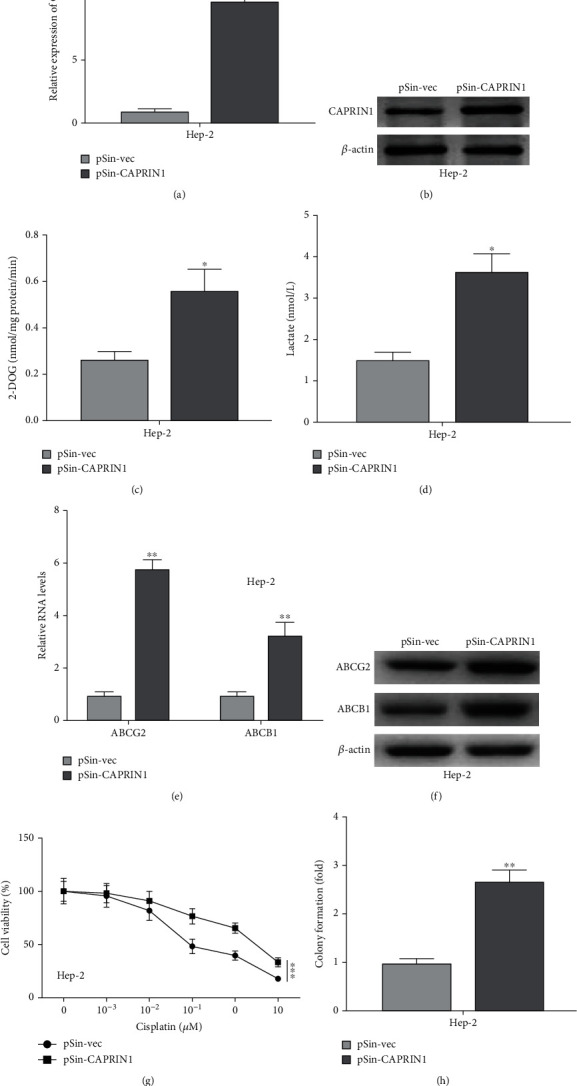
Forced expression of CAPRIN1 promoted glycolysis and chemoresistance in laryngeal cancer cells. (a and b) qRT-PCR and Western blotting were used to determine the mRNA and protein expression levels of CAPRIN1, respectively, in Hep-2 cells that were transfected with CAPRIN1 plasmid (pSin-CAPRIN1) or empty vector (pSin-vec). (c and d) The cellular uptake of 2-DOG and lactate secretion were also determined in those transfected Hep-2 cells. (e and f) qRT-PCR and Western blotting were used to determine the mRNA and protein expression levels of multidrug transporter expression (ABCG2 and ABCB1), respectively, in Hep-2 cells that were transfected with CAPRIN1 plasmid (pSin-CAPRIN1) or empty vector (pSin-vec). (g and h) Cell viability and colony formation were determined to evaluate cell viabilities in cisplatin-treated Hep-2 cells that were transfected with CAPRIN1 plasmid (pSin-CAPRIN1) or empty vector (pSin-vec). The data were shown as the means ± SD. ^∗^*P* < 0.05, ^∗∗^*P* < 0.01, and ^∗∗∗^*P* < 0.001.

We further investigated the roles of CAPRIN1 in glycolysis and chemoresistance. First of all, we constructed CAPRIN1 overexpressing stable cells in Hep-2 cells. The mRNA and protein levels of CAPRIN1 were increased in the pSin-CAPRIN1 transfected Hep-2 cells, indicating that CAPRIN1 overexpressing stable cells were successfully constructed (Figures 2(a) and 2(b)). Interestingly, the levels of 2-DOG and lactate in pSin-CAPRIN1 transfected cells were significantly increased as compared to those pSin-vec transfected cells (Figures 2(c) and 2(d)). Both mRNA and protein levels of ABCG2 and ABCB1 were enhanced in pSin-CAPRIN1 transfected cells (Figures 2(e) and 2(f)). In addition, cell viability assays showed that cisplatin-treated CAPRIN1 overexpressing Hep-2 cells exhibited higher cell viabilities and numbers of colonies as compared to those normal Hep-2 cells (Figures 2(g) and 2(h)), indicating CAPRIN1 overexpressing promoted cisplatin resistance in laryngeal cancer cells.

**Figure 3:**
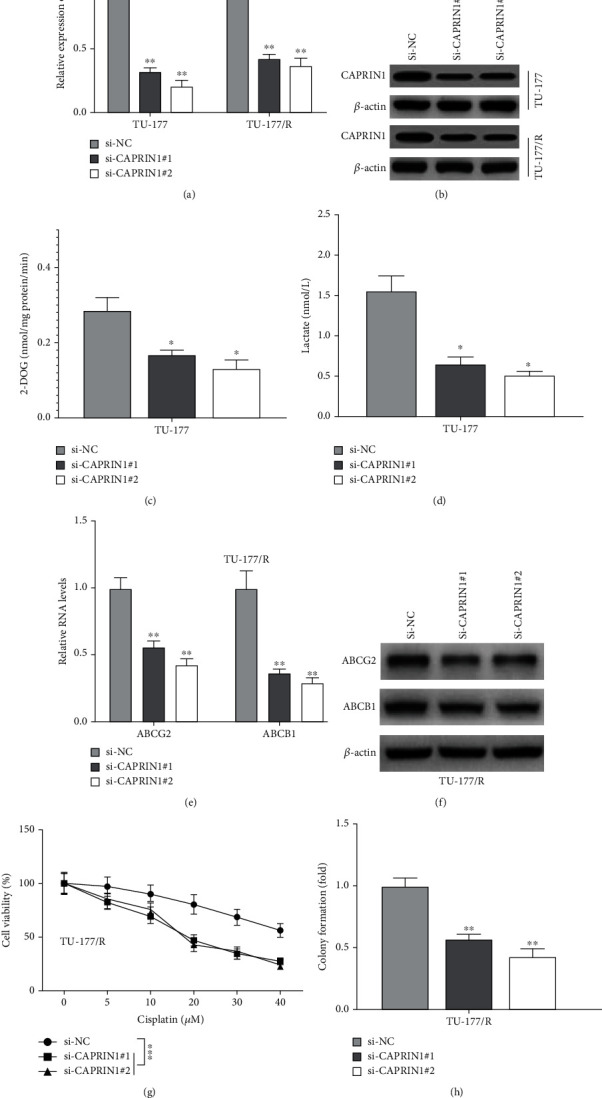
Knockdown of CAPRIN1 inhibited glycolysis and chemoresistance in laryngeal cancer cells. (a and b) qRT-PCR and Western blotting were used to determine the mRNA and protein expression levels of CAPRIN1, respectively, in TU-177 and TU-177/R cells that were transfected with CAPRIN1 siRNAs (si-CAPRIN1#1 and si-CAPRIN1#2) or negative control (si-NC). (c and d) Besides, the cellular uptake of 2-DOG and lactate secretion were also determined. (e and f) qRT-PCR and Western blotting were used to determine the mRNA and protein expression levels of multidrug transporter expression (ABCG2 and ABCB1), respectively, in TU-177/R cells that were transfected with CAPRIN1 siRNAs (si-CAPRIN1#1 and si-CAPRIN1#2) or negative control (si-NC). (g and h) Cell viability and colony formation assay were determined in cisplatin-treated TU-177/R cells that were transfected with CAPRIN1 siRNAs (si-CAPRIN1#1 and si-CAPRIN1#2) or negative control (si-NC). The data were shown as the means ± SD. ^∗^*P* < 0.05, ^∗∗^*P* < 0.01, and ^∗∗∗^*P* < 0.001.

To confirm the roles of CAPRIN1 in glycolysis and chemoresistance, we then knocked down CAPRIN1 in other two laryngeal cancer cell lines, TU-177 and TU-177/R cells. Our results showed that the mRNA and protein levels of CAPRIN1 were decreased in the TU-177 and TU-177/R cells (Figures 3(a) and 3(b)), indicating that CAPRIN1 was successfully silenced. Next, we found that the levels of 2-DOG and lactate were significantly decreased in those CAPRIN1 knockdown cells (Figures 3(c) and 3(d)), indicating that CAPRIN1 knockdown inhibited glycolysis. We also detected the levels of ABCG2 and ABCB1. Our results showed that the mRNA and protein levels of ABCG2 and ABCB1 were decreased in those CAPRIN1 knockdown cells (Figures 3(e) and 3(f)). Furthermore, cell viability assays demonstrated that the cell viability and colony number of TU-177/R cells were significantly decreased in the treatment of cisplatin (Figures 3(g) and 3(h)), indicating that CAPRIN1 knockdown suppressed cisplatin resistance in laryngeal cancer cells.

### 3.3. CAPRIN1 Regulated the ZIC5 Expression

**Figure 4:**
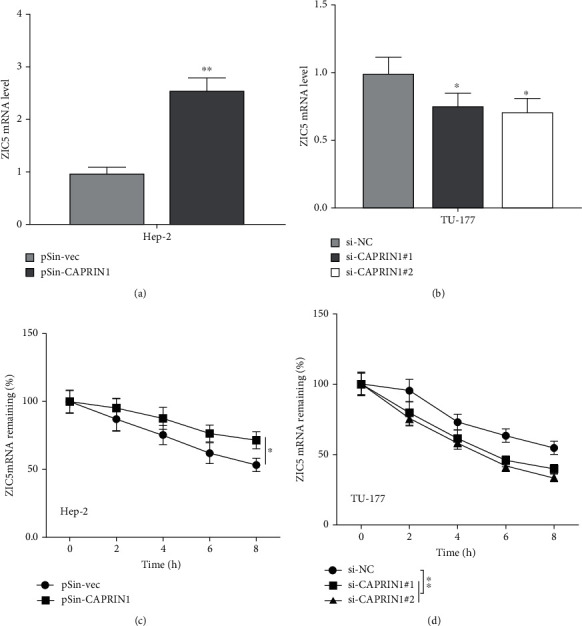
CAPRIN1 regulated the ZIC5 expression. (a and b) qRT-PCR was used to determine the mRNA expression levels of ZIC5 in Hep-2 cells that were transfected with CAPRIN1 plasmid (pSin-CAPRIN1) or empty vector (pSin-vec) and mRNA expression levels of ZIC5 in TU-177 cells that were transfected with CAPRIN1 siRNAs (si-CAPRIN1#1 and si-CAPRIN1#2) or negative control (si-NC). In addition, (c and d) after the cells were transfected for 48 h, the cells were then treated with actinomycin D (10 *μ*g/ml). These cells were harvested at 0, 2, 4, 6, and 8 h after treatment. The mRNA expression levels of ZIC5 were then detected using qRT-PCR. The data were shown as the means ± SD. ^∗^*P* < 0.05 and ^∗∗^*P* < 0.01.

We explored the target of CAPRIN1 by using starBase (http://starbase.sysu.edu.cn/starbase2/browseNcRNA.php) and identified that CAPRIN1 existed binding ability on the mRNA region of ZIC5. To confirm this prediction, we investigated the ZIC5 mRNA expression levels in CAPRIN1 overexpressing and knockdown cells. Interestingly, we found that the mRNA expression levels of ZIC5 were significantly increased in CAPRIN1 overexpression cells and significantly decreased in CAPRIN1 knockdown cells (Figures 4(a) and 4(b)). To confirm this phenomenon, we incubated the cells with actinomycin D, an inhibitor of RNA polymerase. Interestingly, the results showed that a higher percentage of ZIC5 mRNA remains in CAPRIN1 overexpression cells and a lower percentage of ZIC5 mRNA remains in CAPRIN1 knockdown cells (Figures 4(c) and 4(d)). These results suggested that CAPRIN1 regulated the ZIC5 expression.

### 3.4. CAPRIN1 Regulated ZIC5 through 3′UTR Binding

**Figure 5:**
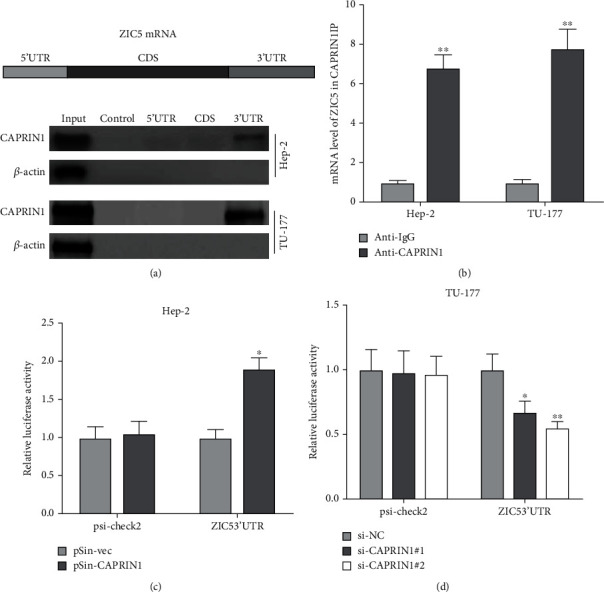
CAPRIN1 regulated ZIC5 through 3′UTR binding. (a) A biotinylated RNA pull-down assay was performed. The biotinylated different regions of ZIC5 mRNA were incubated with cell lysates (Hep-2 and TU-177) and the mRNA region that was interacted with CAPRIN1 was analyzed by using Western blotting. *β*-Actin was used as an internal control. Besides, (b) RNP IP assay was also operated. After mRNA of ZIC5 in cell lysates were captured by anti-CAPRIN1 antibody or anti-IgG, qRT-PCR was then determined. (c) Luciferase reporter activity was performed in the Hep-2 cells that were cotransfected with pSin-CAPRIN1/pSin-vec and ZIC5 3′UTR/psi-check2, respectively. (d) Luciferase reporter activity was detected in the TU-177 cells that were cotransfected with si-CAPRIN1#1/si-CAPRIN1#2/si-NC and ZIC5 3′UTR/psi-check2, respectively.

We further investigated the interactive manner between CAPRIN1 and ZIC5. Biotinylated RNA pull-down assay demonstrated that CAPRIN1 interacted with the 3′UTR region of ZIC5 (Figure 5(a)). Consistently, RNA IP assay showed that mRNA of ZIC5 can be captured by anti-CAPRIN1 antibody (Figure 5(b)), indicating the interactions between CAPRIN1 and ZIC5. To confirm the interaction between CAPRIN1 and ZIC5 3′UTR, we performed the luciferase reporter assays. Interestingly, the cells that cotransfected with pSin-CAPRIN1 plus ZIC5 3′UTR exhibited higher luciferase activities (Figure 5(c)). However, the cells that cotransfected with siCAPRIN1 (siCAPRIN#1 and siCAPRIN#2) plus ZIC5 3′UTR exhibited lower luciferase activities (Figure 5(d)). Taken together, these results supported that CAPRIN1 regulated ZIC5 through 3′UTR binding.

### 3.5. CAPRIN1 Promoted Glycolysis and Chemoresistance of Laryngeal Cancer Cells by the Regulation of ZIC5

**Figure 6:**
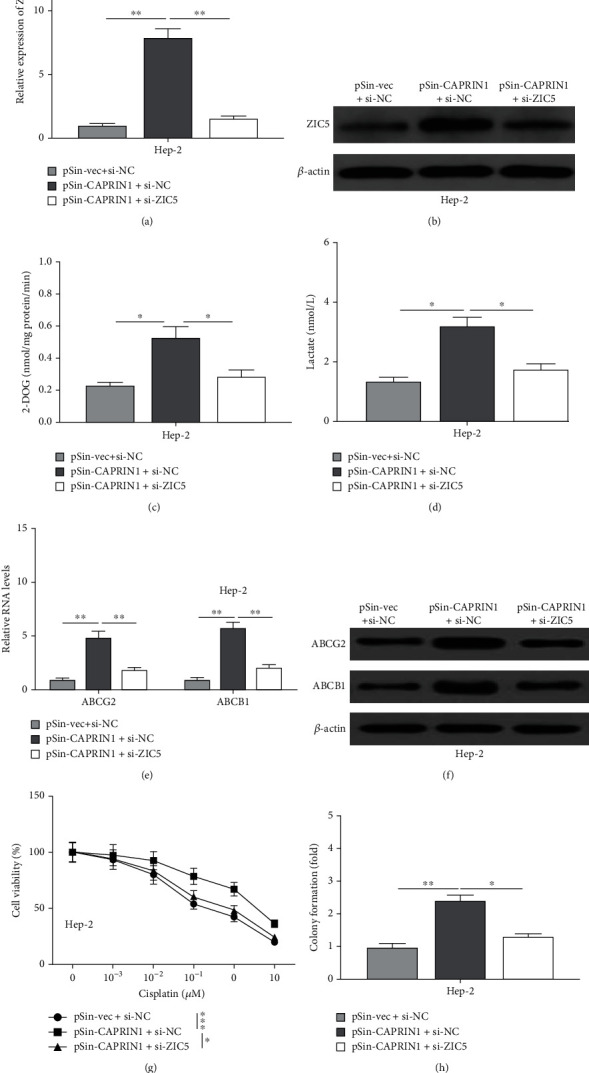
CAPRIN1 promoted glycolysis and chemoresistance of laryngeal cancer cells by the regulation of ZIC5. Hep-2 cells were transfected with pSin-vec plus si-NC, pSin-CAPRIN1 plus si-NC, or pSin-CAPRIN1 plus si-ZIC5. (a and b) qRT-PCR and Western blotting were used to determine the mRNA and protein expression levels of ZIC5. (c and d) Besides, the cellular uptake of 2-DOG and lactate secretion were determined. (e and f) qRT-PCR and Western blotting were used to determine the mRNA and protein levels of multidrug transporter expression (ABCG2 and ABCB1), respectively. (g and h) CCK-8 and colony formation assays were performed to determine cell viability in cisplatin-treated Hep-2 cells that were transfected. The data were shown as the means ± SD. ^∗^*P* < 0.05, ^∗∗^*P* < 0.01, and ^∗∗∗^*P* < 0.001.

Finally, we investigated whether the regulatory effects of CAPRIN1 on glycolysis and chemoresistance are associated with ZIC5. First, Hep-2 cells were cotransfected with pSin-vec plus si-NC, pSin-CAPRIN1 plus si-NC, or pSin-CAPRIN1 plus si-ZIC5. We found that mRNA and protein levels of ZIC5 were significantly increased in cells that were transfected pSin-CAPRIN1 plus si-NC (Figures 6(a) and 6(b)), indicating that CAPRIN1 overexpression promoted the ZIC5 expression. Interestingly, we observed that the levels of 2-DOG and lactate in CAPRIN1 overexpression cells were significantly increased, whereas ZIC5 knockdown was accompanied by the decrease of 2-DOG and lactate (Figure 6C–6D). We also detected the levels of ABCG2 and ABCB1. Our results showed that the levels of ABCG2 and ABCB1 in CAPRIN1 overexpression cells were significantly increased, whereas ZIC5 knockdown accompanied by the decrease of ABCG2 and ABCB1 (Figures 6(e) and 6(f)). In addition, we also observed that cells with CAPRIN1 overexpression exhibited higher cell viability and colony number, whereas ZIC5 knockdown inhibited cell viability and colony number (Figures 6(g) and 6(h)).

**Figure 7:**
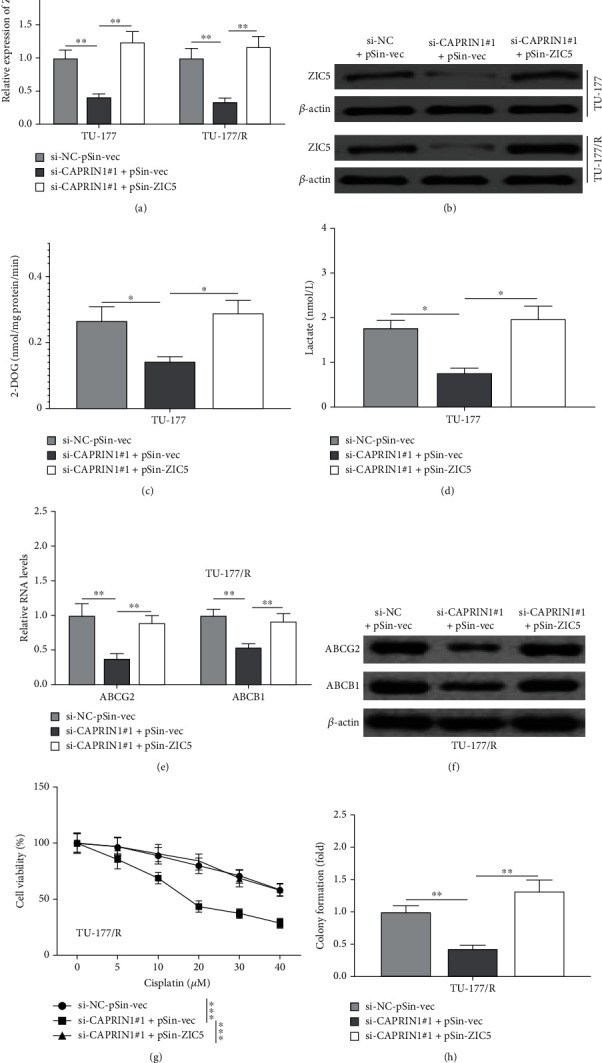
Effects of CAPRIN1 on glycolysis and chemoresistance of laryngeal cancer cells depended on ZIC5. TU-177 and TU-177/R cells were also transfected with si-NC plus pSin-vec, si-CAPRIN1#1 plus pSin-vec, or si-CAPRIN1#1 plus pSin-ZIC5. (a and b) The mRNA and protein expression levels of ZIC5 in those transfected cells were determined. (c and d) The cellular uptake of 2-DOG and lactate secretion were determined. (e and f) qRT-PCR and Western blotting were used to determine the mRNA and protein expression levels of multidrug transporter expression (ABCG2 and ABCB1), respectively. (g and h) Cell viability of these cotransfected TU-177/R cells after 24-h treatment with the indicated dose of cisplatin was determined by using MTT assay and colony formation assay, respectively. The data were shown as the means ± SD. ^∗^*P* < 0.05, ^∗∗^*P* < 0.01, and ^∗∗∗^*P* < 0.001.

Furthermore, to confirm the regulatory effects of CAPRIN1 on glycolysis and chemoresistance associated with ZIC5. TU-177 and TU-177/R cells were transfected with si-NC plus pSin-vec, si-CAPRIN1#1 plus pSin-vec, or si-CAPRIN1#1 plus pSin-ZIC5. We observed that mRNA and protein levels of ZIC5 were significantly increased in cells that were transfected si-CAPRIN1#1 plus pSin-vec (Figures 7(a) and 7(b)), indicating CAPRIN1 knockdown suppressed the ZIC5 expression. Interestingly, we observed that the levels of 2-DOG and lactate in CAPRIN1 knockdown cells were significantly decreased, whereas ZIC5 overexpression was accompanied by the increase of 2-DOG and lactate (Figures 7(c) and 7(d)). The levels of ABCG2 and ABCB1 were decreased, whereas ZIC5 overexpression was accompanied by the elevation of ABCG2 and ABCB1 (Figure 7E–7F). Besides, we also observed that cells with CAPRIN1 knockdown exhibited lower cell viability and colony number, whereas ZIC5 overexpression enhanced cell viability and colony number (Figures 7(g) and 7(h)). Taken together, these results suggested that CAPRIN1 promoted glycolysis and chemoresistance of laryngeal cancer cells by the regulation of ZIC5.

## 4. Discussion

In this study, we identified that CAPRIN1 was upregulated in the laryngeal cancer tissues and associated with poor prognosis in patients with laryngeal cancer. Interestingly, upregulated CAPRIN1 promoted glycolysis and cisplatin resistance, whereas CAPRIN1 knockdown suppressed glycolysis and cisplatin resistance in laryngeal cancer cells. ZIC5 was identified as a target of CAPRIN1, which was supported by the interactions between CAPRIN1 and ZIC5 3′ UTR. The underlying mechanism investigation revealed that CAPRIN1 promoted glycolysis and chemoresistance in laryngeal cancer cells was mediated by its interaction with ZIC5. These results supported that targeting CAPRIN1 might be an effective strategy for laryngeal squamous cell carcinoma therapy.

It is known that CAPRIN1 is associated with cell proliferation and migration-related genes and is involved in the tumorigenesis process (18, 20). In addition, CAPRIN1 is highly expressed when tumor cells adapt the adverse conditions, thereby promoting chemoresistance. Gong and colleagues reported that overexpression of CAPRIN1 promotes breast cancer cell proliferation and invasion (26). In another study, Lu and colleagues found that CAPRIN1 promotes cell proliferation, invasion and metastasis, whereas inhibition of CAPRIN1 suppresses the development of gastric cancer (21). However, the roles of CAPRIN1 in laryngeal cancer are still unclear. In this study, we found that CAPRIN1 was highly expressed in laryngeal cancer tissues as compared to adjacent normal tissues. Interestingly, high expression of CAPRIN1 was associated with patients with laryngeal cancer. These results are consistent with a previous study. Tan and colleagues reported that CAPRIN1 is highly expressed in hepatoma cancer tissues as compared to the adjacent normal tissues and is associated with poor survival rates in patients with hepatoma (20). In addition, we also found an elevation of mRNA and protein expressions in laryngeal cancer lines. These results supported the oncogenic roles of CAPRIN1 in laryngeal cancer.

Laryngeal squamous cell carcinoma is featured by distinctive metabolic profiles including aerobic glycolysis (7). A high rate of glycolysis leads to the production of lactate and a highly acidic tumor microenvironment (7, 8). Besides, it also regulates the expression of oncogenes and activates hypoxia-inducible factor-1, resulting in the laryngeal squamous cell carcinoma cell growth and metastasis (8, 9). In this study, we further investigated whether the roles of CAPRIN1 in laryngeal cancer were associated with glycolysis. Interestingly, we found that overexpression of CAPRIN1 led to high levels of 2-DOG and lactate, whereas CAPRIN1 knockdown reversed the production of 2-DOG and lactate. These results indicated that CAPRIN1 promoted glycolysis in laryngeal cancer.

Cisplatin is widely used in patients with advanced laryngeal cancer or who received surgery at recurrence (27). Cisplatin therapy achieves satisfactory effects in the initial stage (27). Unfortunately, patients who initially respond to cisplatin therapy often developed acquired drug resistance after long-term exposure to cisplatin (27, 28). In this study, we further investigated the relationship between CAPRIN1 and cisplatin resistance. The results demonstrated that knockdown of CAPRIN1 suppressed cell viability of laryngeal cancer cell lines that were treated by cisplatin, indicating that CAPRIN1 knockdown suppressed cisplatin-resistance in laryngeal cancer cells. These results suggested that targeting CAPRIN1 might be a good strategy for mitigating cisplatin resistance in the treatment of laryngeal cancer.

In this study, ZIC5 was identified as a target of CAPRIN1 in laryngeal cancer. It is known that ZIC5 belongs to the Zinc family, which is responsible for the regulation of the cell cycle (29). An elevation of ZIC5 was frequently observed in a series of cancer types including glioma, hepatoma, non-small-cell lung cancer, and prostate cancer (30–32). We found that CAPRIN1 regulated the expression of ZIC5 through 3′UTR binding. Moreover, to confirm that the oncogenic roles of CAPRIN1 are mediated by ZIC5, we constructed a CAPRIN1 knockdown cell line in the presence or absence of pSin-ZIC5. Interestingly, we found that CAPRIN1 knockdown resulted in lower cell viability in the presence of cisplatin, whereas ZIC5 overexpression increased cell viability. The levels of 2-DOG and lactate were significantly decreased in CAPRIN1 knockdown cells, whereas ZIC5 overexpression led to the increase of 2-DOG and lactate. Taken together, these results suggested that CAPRIN1 promoted glycolysis and chemoresistance in laryngeal cancer cells by regulation of ZIC5.

## 5. Conclusion

An elevation of CAPRIN1 expression was associated with chemoresistance. In addition, high expression of CAPRIN1 was associated with poor prognosis in patients with laryngeal cancer. The regulatory effects of CAPRIN1 on glycolysis and chemoresistance of laryngeal cancer cells were correlated to its interactions with the ZIC5 3′UTR region.

## Data Availability

Further enquiries of the datasets supporting the results of this article can be directed to the corresponding author.
